# Human Anatomy

**Published:** 1849-11

**Authors:** 


### ARTICLE IX.

*Human Anatomy.*—By Jones Quain, M.D. Edited by Richard Quain, F.R.S., and William Sharpey, M.D. F.R.S., Professors of Anatomy and Physiology, in University College, London. First American, from the Fifth London Edition. Edited by Joseph Leidy, M.D. With over Five Hundred Illustrations. Philadelphia: Lea & Blanchard. 1849. 2 vols. 8 vo. pp. 1,277. (From the Publishers. For sale by Jos. Keen & Bro., Chicago.)

This is the second large and valuable work on Anatomy which we have had the pleasure of introducing to the notice of our readers within the year. In this department of science, the student has now a great variety from which to make a selection. In some particulars the present work has advantages over any other. The section devoted to General Anatomy is very extensive and valuable. The description of the arteries is very full, and their variations from the normal mode of distribution is always noted, as well as their surgical relations. The treatise on Osteology is more complete than is to be found in any other work on Anatomy, embracing, as it does, the history of the development of each bone of the skeleton. The muscles, however, though well treated of, are not described with so much minuteness as in the valuable little work of Von Behr.

To those who wish an extensive treatise on Anatomy, we recommend these handsome volumes as the best that have ever issued from the English or American Press. While saying this, we do not by any means wish to undervalue the many other excellent works in this department. As Americans, we feel proud of the excellent and beautiful volume of Dr. Morton, and the works of Profs. Horner and Wistar; all of which are in some respects better for students than the volumes before us. But as a full and systematic work on Anatomy, we apprehend that Quain and Sharpey's Anatomy will come to be regarded as superior to any thing that has preceded it.

As may be supposed, but little was left for the American editor to supply. What he has done, however, is done well. The eminent publishers who have brought out the work, have executed their task in such a manner as to leave nothing to wish for.

We could not give our readers a correct idea of the real value of the work, without furnishing an elaborate analysis.— This our limits forbid. As an illustration of the style in which the work is written, and the fullness of the details with which it abounds, we quote the following paragraph from the section on Osteology, which we are inclined to regard as the most valuable portion of the work:

### *Analogy between Cranial Bones and Vertebræ.*

"Anatomists have at all times perceived and recognised the analogy between the movable and motionless pieces of the spine—between those of the lumbar and dorsal regions, and those of the sacrum and coccyx; in the one, as well as in the other, similar organic developments are observed to exist, variously modified, in order to suit special purposes; but it is only of late years that any adequate attention has been directed to the points of similitude which exist between vertebræ, properly so called, and the cranial bones. Many persons who adopt, without hesitation, the terms false or pelvic vertebræ, as applied to the sacrum and coccyx, feel a repugnance to use false or cranial, as applied to the pieces of the skull; and deny, perhaps, without examination, the analogy upon which it is founded, as being unnatural or far-fetched. We have numerous instances of the harmony that subsists between containing and contained parts throughout the economy; in no case is it more striking than in the relation that obtains between the fundamental part of the osseous structure and the central mass of the nervous system. The spinal canal is accurately adapted in its different parts to the nervous cord which it encloses. In the pelvic region, the canal, at least in the human subject, becomes narrow, as it merely encloses nerves, whilst the body and processes take on a particular development to meet a special purpose, that of forming a basis of support for the rest of the column. This seems to result from the working of what may be termed a principle of compensation in the growth as well as in the action of parts; for when one part of a given whole is developed to excess or to a maximum, others will remain at a minimum or atrophied: thus the spinal canal and the arches are at their minimum in the sacrum and coccyx, for the contained parts are there at a low point of development; but at the opposite end of the column the reverse obtains; the contained parts, viz: the central parts of the nervous system, are evolved in the human subject to the greatest extent, and so must the containing parts also be. The portion of the osseous system which corresponds with the bodies of the vertebræ can, therefore, hardly be recognised; whilst that which is analogous to the arches is expanded so much as to retain but a slight smilitude to them.

If we take the occipital bone, and examine it attentively, we shall readily perceive in it all the elements of a vertebra. The foramen magnum is the counterpart of the ring of a vertebra, and has a similar relation to the spinal cord; the basilar process represents the body; the condyles are true articulating processes; the rough surfaces external to them, and which give attachment to the recti laterales, correspond to the transverse processes; the vertical ridge extended backwards along the median line, from the foramen to the occipital protuberance, is, in the human subject, merely a rudiment of a spinal process; but in the dog, bear, and badger, it forms a sharp prominence well deserving the name of spine, and the likeness is still more striking in osseous fishes: finally, the broad plates on each side of the spine represent the arches. In this view of the matter, the occipital bone forms the first false vertebra of the cranial region.

"In the second cranial piece or vertebra, it must be admited that the analogies are not so striking; but when we recollect that the cavity of the skull, if examined in the different orders of animals, enlarges in proportion as the brain acquires an increase of development, and that this enlargement attains its maximum in the human subject, we shall at once find sufficient reason to expect that the parts corresponding with the vertebral arches should, in this region, be greatly evolved, while the rest are in a manner atrophied. The parietal bones, with the squamous part of the temporal and the great wings of the sphenoid, taken together, represent the arches, whilst the posterior part of the sphenoid bone, (such as it exists in the human fœtus before its ossification is complete, and such as it continues permanently in several lower animals,) is the counterpart of the body, the mastoid processes of the temporal bones with the glenoid fossæ serve as transverse and ariculating processes. These, together, form the middle cranial piece, which may be termed the spheno-temporo-parietal cranial false vertebra.

"The frontal bone, the ethmoid, and the anterior division of the sphenoid, (which is that part of the body that sustains the smaller wings,) from the third vertebra; the part of the sphenoid just named, together with the crista galli and the perpendicular plate of the ethmoid bone, form the body, which is here reduced to a rudimentary state, just as the coccygeal bones are at the opposite end of the column, of which it may be considered a repetition. The lateral and expanded parts of the frontal bone are the arches, and the external orbital processes may be likened to transverse processes.

We have here used the term false vertebra as applied to the cranial pieces; perhaps it would be better to use the word zone, as sanctioned by the authority of Cuvier. The passage in which he recognises the principle of the development here indicated, as well as the application of it, (which appears to have been first inculcated by Dumeril, and traced in all its details by Geoffroy Saint-Hilaire) is as follows:—'Le crâne se subdivise comme en trois ceintures, formèes—l'antèrieure par les deux frontaux et l'èthmoide, l'intermèdiare par les pariétaux et le sphènoide, la postèrieure par l'occipital.'"*

